# Diagnostic performance of cytomegalovirus (CMV) immune monitoring with ELISPOT and QuantiFERON-CMV assay in kidney transplantation: A PRISMA-compliant article

**DOI:** 10.1097/MD.0000000000015228

**Published:** 2019-04-19

**Authors:** Yashi Ruan, Wei Guo, Sudong Liang, Zhen Xu, Tianli Niu

**Affiliations:** Department of Urology, Taizhou People's Hospital, Jiangsu, China.

**Keywords:** cytomegalovirus, immune monitoring, kidney transplantation, meta-analysis, systematic review

## Abstract

**Background:**

Cytomegalovirus (CMV) infection is part of major infection complications following kidney transplantation. However, more rapid and low-complexity assays are needed for CMV infection. Our study is to investigate the diagnostic efficacy of 2 novel tests, CMV-ELISPOT and QuantiFERON-CMV tests, in CMV DNA viremia and CMV infection following renal transplant.

**Methods:**

We searched MEDLINE, EMBASE, the Cochrane Central Register of Controlled Trials and the Web of Science. Case–control or cohort study designed to explore the CMV-ELISPOT and/or QuantiFERON-CMV tests in the recipients with CMV infection was considered to be eligible for this study. Sensitivity (SEN), specificity (SPE), diagnostic odds ratio (DOR), and summary receiver-operating characteristic (SROC) curves were calculated.

**Results:**

We selected a total of 12 articles for systematic review and 11 of them were included in meta-analysis. For CMV-pp65 assay, the pooled SEN, SPE, and DOR were 0.73 (95% confidence interval [CI], 0.67–0.78), 0.61 (95% CI, 0.56–0.65), and 4.46 (95% CI, 3.11–6.39), respectively. For CMV-IE-1 assay, the pooled SEN, SPE, and DOR were 0.84 (95% CI, 0.78–0.88), 0.46 (95% CI, 0.42–0.51), and 5.07 (95% CI, 3.26–7.89), respectively, whereas the pooled SEN, SPE, and DOR of QuantiFERON-CMV test were 0.38 (95% CI, 0.28–0.49), 0.38 (95% CI, 0.32–0.44), and 1.02 (95% CI, 0.17–6.00).

**Conclusions:**

We reported that CMV-ELISPOT tests, including CMV-pp65 and CMV-IE-1, perform well in the diagnosis and prediction of CMV infection in renal transplant recipients, whereas QuantiFERON-CMV test needs further exploration.

## Abstract 2

Supplemental Digital Content is available in the text

## 1 Introduction

Although remarkable improvements in the management and treatment of cytomegalovirus (CMV) infection have been achieved, human CMV infection still remains to be one of major infection complications following kidney transplantation.^[1]^ In renal transplant recipients, CMV-mediated indirect effects on organ damage include chronic allograft nephropathy and allograft rejection.^[2,3]^ Among these renal donors and allograft recipients, seropositivity has been recognized as a biomarker for latent CMV infection. Recipients with highest risk of CMV infection are the combination of a CMV seronegative recipients and a CMV seropositive donor (D+/R−), which represent 15% to 25% in all renal transplant recipients.^[4]^ Moreover, 5% of CMV seronegative recipients with CMV seronegative donors (D−/R−) also suffer from posttransplant CMV infection, mainly due to the social contacts and blood transfusion.^[2,5]^ Thus, accurate identification of the risks of renal transplant recipients with CMV infection is still urgently needed in clinical practice.

In recent years, several studies reported that the recovery of CMV-specific CD4+ and CD8+ T cells is related to the long-term protection from CMV reactivation and viremia, and reduced CMV-mediated damage.^[6–8]^ Therefore, monitoring the CMV-specific cellular immunity has been investigated to better predict the risk of CMV infection among recipients.^[9]^ In general, the most commonly used diagnostic tools based on the principle of CMV-specific cellular immunity include CMV enzyme-linked immunospot (ELISPOT) assays and QuantiFERON-CMV test. The T-cell immune activity of former one could be measured by assessing the interferon-gamma (IFN-γ) production after the stimulation with CMV antigens such as phosphoprotein 65 (pp65) and immediate early 1 (IE-1), whereas the latter one is an enzyme-linked immunosorbent assay (ELISA) tool to test the released levels of IFN-γ in the whole blood by ex vivo stimulation with human leukocyte antigen (HLA) class I-restricted CMV peptides.^[10–12]^ However, controversial conclusions with regard to these novel technologies were observed across studies.

In our study, we performed the comprehensive systematic review to compare various studies investigating the CMV ELISPOT and/or QuantiFERON-CMV assays, and carried out the meta-analysis to quantitatively evaluate the diagnostic performance of these assays in CMV DNA viremia and infection following kidney transplantation.

## 2 Methods and materials

### 2.1 Ethics statement

The protocols followed in this study were in accordance with the ethical standards of the Declarations of Helsinki and Istanbul, and approved by the local ethics committee of Taizhou People's Hospital.

### 2.2 Search strategy

The following databases were comprehensively searched for studies published between January 1, 1990 and February 1, 2018: MEDLINE, EMBASE, the Cochrane Central Register of Controlled Trials (CENTRAL) and Web of Science (WOS). The search was performed using the following search keywords in combination: (“CMV” OR “cytomegalovirus”) AND (“Enzyme-Linked Immunospot Assay” OR “ELISPOT” OR “QuantiFERON” OR “QF”) AND (“kidney transplantation” [MeSH]). In addition, the reference lists of all articles in eligible studies were also read to identify any additional relevant literature.

### 2.3 Inclusive and exclusive criteria

Eligible studies have to meet the following criteria: case–control or cohort study with prospective or retrospective; sufficient data for pooled analysis (true positives [TP], true negatives [TN], false positives [FP], and false negatives [FN]) to predict viremia; if data or subsets of data were used in series trials, the more recent article, or the one with more detail was chosen; and articles written in English or Chinese. The exclusion criteria included the following: reviews, case reports, and letters to editors; duplicate publications; studies in languages other than English or Chinese; and studies with insufficient data to construct a 2 × 2 table. Two reviewers (YSR and WG) independently reviewed the eligible studies. Consensus was reached for each eligible study and any disagreements were resolved by consultation with a third reviewer (TLN).

### 2.4 Data extraction and management

Two authors (YSR and WG) independently extracted data for all eligible studies, including author, publication year, study design, number of participants included, ethnicity, male/female, average age, positive predictive and negative predictive value, FP and FN predictive values of DNA viremia of CMV ELISPOT assay, or QuantiFERON-CMV test.

### 2.5 Quality assessment

We used the Quality Assessment of Diagnostic Accuracy Studies tool (QUADAS-2) to evaluate the risk of bias in 4 domains, which included patient selection, index test, reference standard, as well as flow and timing.^[13]^ According to the QUADAS-2 guidelines, 2 authors (YSR and WG) assessed risk of bias for these 4 domains as low, high, or unclear. In case of disagreement, we resolved by discussion with a third author (TLN).

### 2.6 Statistical analysis

The meta-analysis was carried out by pooling the sensitivity (SEN), specificity (SPE), positive likelihood ratios (PLR), negative likelihood ratios (NLR), and diagnostic odds ratios (DOR) with random effects models and their 95% confidential intervals (CIs) for the overall CMV tests, CMV-IE-1, CMV-pp65, and QuantiFERON-CMV tests, to evaluate the risk of DNA viremia. Moreover, the corresponding summary receiver-operating characteristic (SROC) curves were calculated to express the test parameter results. The AUC-ROCs were always close to 1 when a test was accurate, and in contrast, poor tests usually had an AUC-ROC approaching 0.5.^[14]^

In addition, heterogeneity among these studies was quantitatively assessed with the inconsistency index (*I*^2^), which indicated substantial heterogeneity if the value of *I*^2^ was >50%. We calculated Spearman's correlation coefficient to assess the potential threshold effect. All of the statistical analyses were conducted using Meta-Disc software (version 1.4; the Ramón y Cajal Hospital, Madrid, Spain).^[15]^

## 3 Results

### 3.1 Study selection and basic characteristics

**Figure 1:**
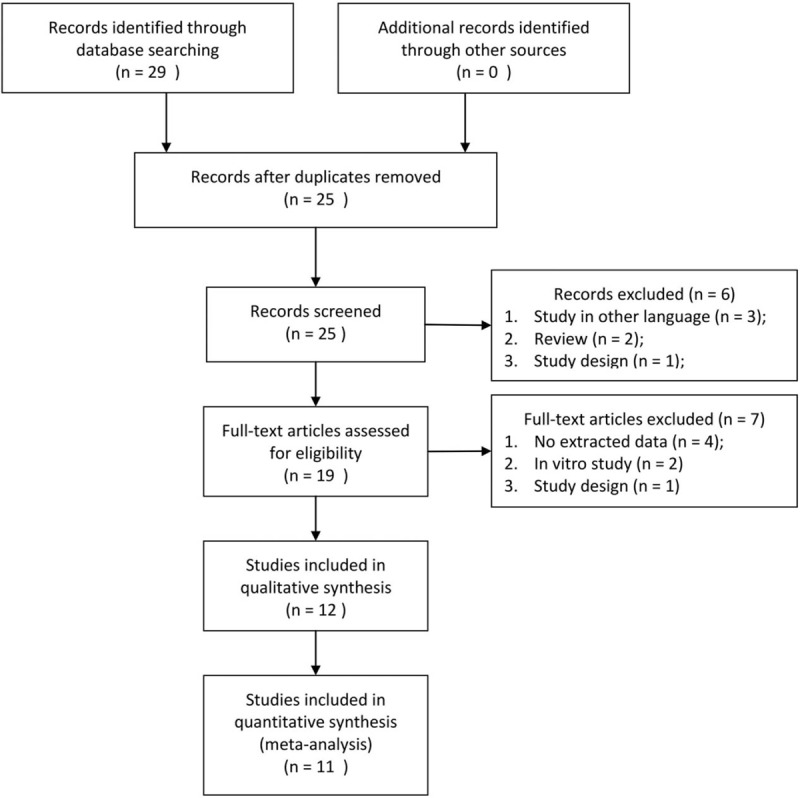
PRISMA flow diagram.

We identified a total of 25 studies after the original literature search (Fig. 1). After screening the titles and abstracts, 6 studies were deleted according to the exclusive criteria. In general, 12^[16–27]^ studies were included in our systematic review, and 11 of which were selected for quantitative analysis.

**Figure 2:**
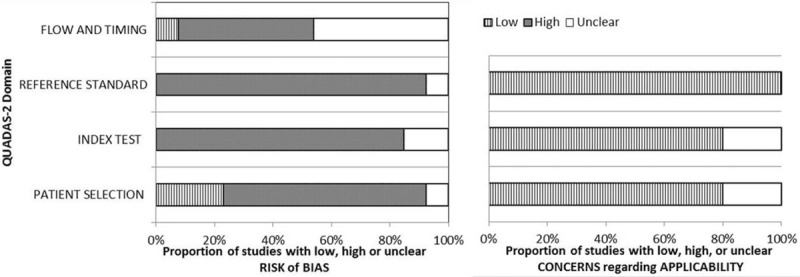
Results of quality assessment across studies evaluated by QUADAS-2.

The results of systematic review are presented in Table 1. The primary outcomes of all eligible studies in our study were the diagnostic accuracy of CMV-ELISPOT and/or QuantiFERON-CMV tests, including SEN and SPE, to predict CMV DNA viremia. Nine of 10 studies were carried out in the ethnicity of Caucasian, and 1 study was carried out in Asians. Seven studies evaluated the CMV-pp65 assay, 6 studies evaluated the CMV-IE-1 assay, and 4 studies evaluated the QuantiFERON-CMV test. Importantly, there were 6 studies reported the administration of prophylactic treatment for CMV (−) recipients, and our results of meta-regression analysis did not support the significant impact of prophylactic treatment on the pooled results. The results of quality assessment across studies are shown in Figure 2. The majority of included studies clearly stated the aspects related to 4 domains in QUADAS-2, indicating the overall quality is moderate–high. Moreover, there is no disagreement during the study selection, data extraction, and quality assessment.

**Table 1:**
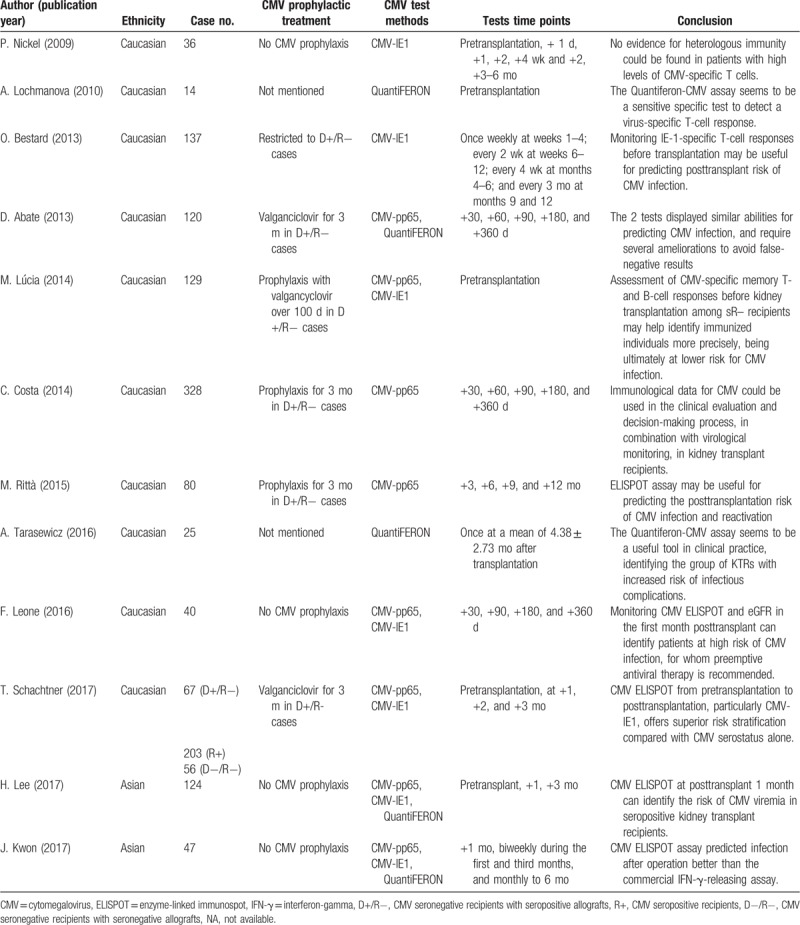
Basic characteristics of eligible studies in systematic review and meta-analysis.

### 3.2 Diagnostic accuracy of CMV ELISPOT and QuantiFERON-CMV tests

**Figure 3:**
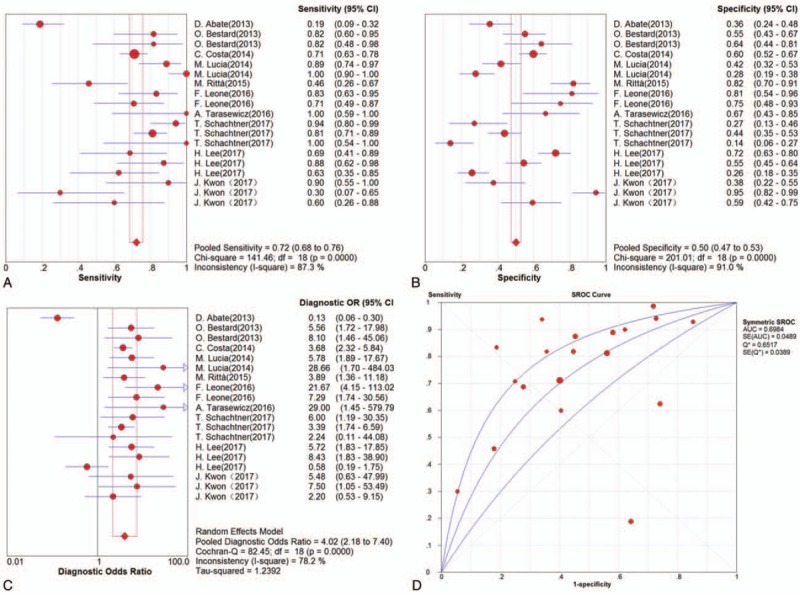
Results of diagnostic meta-analysis for overall CMV immune monitoring in kidney transplantation, including the pooled sensitivity (A), specificity (B), diagnostic odds ratio (C), and SROC curve (D).

Eleven studies with 1167 renal transplant recipients were enrolled in the meta-analysis for all 3 tests (CMV-pp65, CMV-IE-1, and QuantiFERON-CMV). First, we evaluated the threshold effects in the pooled data, and the results showed that Spearman correlation coefficient and its *P* value were 0.43 and 0.067, which suggested no significant threshold effect exists. The pooled SEN and SPE estimates were 0.72 (95% CI, 0.68–0.76) and 0.50 (95% CI, 0.47–0.53) to predict CMV DNA viremia, respectively (Fig. 3A and B). The pooled PLR and NLR estimates were 1.54 (95% CI, 1.32–1.81) and 0.47 (95% CI, 0.32–0.69) (Supplemental Fig. 1A and B), whereas the pooled DOR estimates were 4.02 (95% CI, 2.18–7.40) (Fig. 3C). The AUC estimates of SROC curve were 0.70 (Fig. 3D). Significant heterogeneity was observed in both pooled SEN (*I*^2^ = 87.3%, *P* < 0.001) and SPE (*I*^2^ = 91.0%, *P* < 0.001), and then the random effects model was selected for further analysis. To explore the sources of extreme heterogeneity, we performed the meta-regression based on 2 potential confounding factors, including the ethnicity and methods. The results showed that test methods were significantly associated with relatively high heterogeneity, indicating the subgroup analysis of individual CMV diagnostic methods is necessary (coefficient: 0.94, *P* = 0.017; Supplemental Table 1).

**Figure 4:**
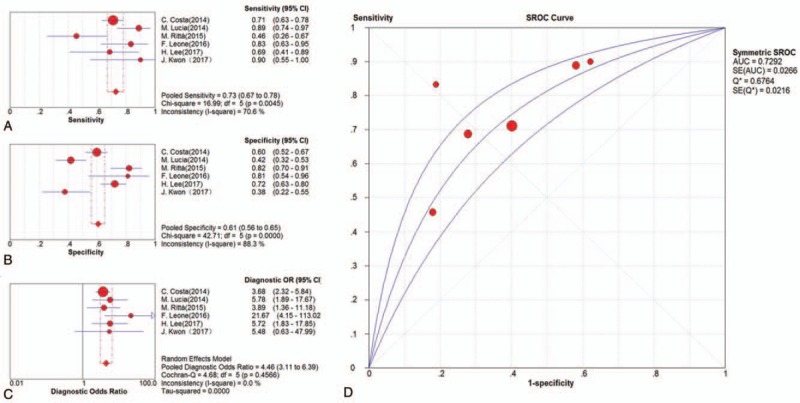
Results of diagnostic meta-analysis for CMV-pp65 assay in kidney transplantation, including the pooled sensitivity (A), specificity (B), diagnostic odds ratio (C), and SROC curve (D).

Six clinical trials were included in the meta-analysis for CMV-pp65 assay. The pooled SEN and SPE estimates were 0.73 (95% CI, 0.67–0.78) and 0.61 (95% CI, 0.56–0.65), respectively (Fig. 4A and B). The pooled PLR and NLR estimates were 1.83 (95% CI, 1.47–2.27) and 0.44 (95% CI, 0.32–0.61) (Supplemental Fig. 1C and D), whereas the pooled DOR estimates were 4.46 (95% CI, 3.11–6.39) (Fig. 4C). The AUC estimates of SROC curve were 0.73 (Fig. 4D).

**Figure 5:**
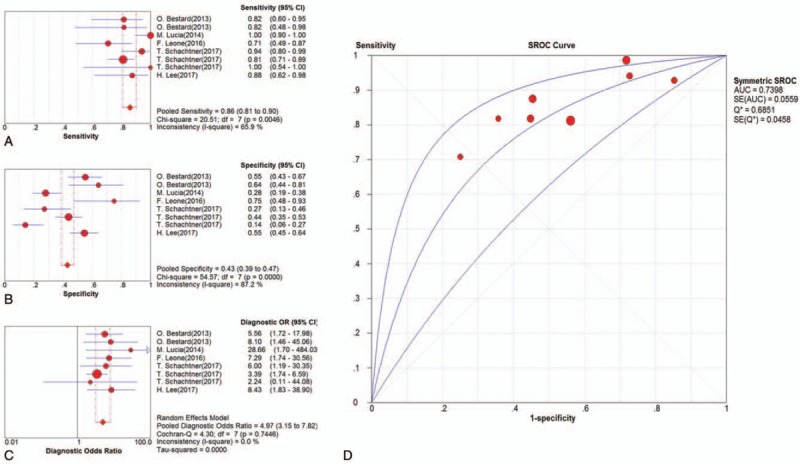
Results of diagnostic meta-analysis for CMV-IE-1 assay in kidney transplantation, including the pooled sensitivity (A), specificity (B), diagnostic odds ratio (C), and SROC curve (D).

Likely, 6 clinical trials were included in the meta-analysis for CMV-IE-1 assay. The pooled SEN and SPE estimates were 0.84 (95% CI, 0.78–0.88) and 0.46 (95% CI, 0.42–0.51), respectively (Fig. 5A and B). The pooled PLR and NLR estimates were 1.54 (95% CI, 1.30–1.82) and 0.39 (95% CI, 0.25–0.60) (Supplemental Fig. 1E and F), whereas the pooled DOR estimates were 5.07 (95% CI, 3.26–7.89) (Fig. 5C). The AUC estimates of SROC curve were 0.75 (Fig. 5D).

Four studies were eligible for quantitative analysis of QuantiFERON-CMV test. The results showed that the pooled SEN and SPE estimates were 0.38 (95% CI, 0.28–0.49) and 0.38 (95% CI, 0.32–0.44) (Supplemental Fig. 2A and B). The pooled PLR and NLR estimates were 1.00 (95% CI, 0.40–2.48) and 1.11 (95% CI, 0.47–2.58) (Supplemental Fig. 2C and D), whereas the pooled DOR estimates were 1.02 (95% CI, 0.17–6.00) (Supplemental Fig. 2E). The AUC estimates of SROC curve were 0.41 (Supplemental Fig. 2F).

## 4 Discussion

This is the first study in English literature to perform a systematic review and meta-analysis of the predictive utility of CMV-immune monitoring assays in kidney transplantation. In this study, we reported that CMV-ELISPOT tests, including CMV-pp65 and CMV-IE-1, perform well in the prediction of CMV infection in renal transplant recipients, whereas QuantiFERON-CMV test needs further exploration.

In the systematic review, favorable conclusions for CMV ELISPOT assays were observed, whereas one study^[22]^ reported no difference in CMV-specific T-cell frequencies between recipients with or without detectable CMV antibodies. However, the authors also found that strong CMV-IE-1-specific T-cells response were significantly associated with the less alloreactivity and improved graft function, indicating that CMV-IE-1-specific T-cells response, which underlined the importance of IE-1 as a target of anti-CMV T cells and were consistent with previous studies.^[28,29]^ Notably, CMV-pp65/IE-1 IFN-γCD8+ and CD4+ T cells enumerated by flow cytometry (FCM) has been explored to investigate the dynamics of CMV-specific T-cells immunity in several transplant centers.^[30–32]^ Among eligible studies in systematic review, the usage of target peptide and methodology may be 2 major confounding factors resulting in the inconsistent conclusions. Two classic peptides, including CMV-pp65 and CMV-IE-1, were utilized to duplicate wells or selectively solo well in ELISPOT assay, and no study reported the difference between these 2 target peptides in the outcomes of assays.^[33]^ Although comparisons of CMV-pp65/IE-1 between ELISPOT and FCM still need further confirmation, it does provide a novel insight to predict the CMV infections in solid organ transplantation.

The results of our meta-analysis did not support the application of QuantiFERON-CMV test in the prediction of CMV infection in renal transplant recipients. It is reported that CMV seropositive recipients with negative cellular mediated immunity tended to be positive in the QuantiFERON-CMV test when stimulated with non-HLA-restricted whole CMV virion lysate, which suggested the potential limitation of the stimuli used in the QuantiFERON-CMV test.^[17]^ Moreover, the immune system would be dynamic when interplayed with positive and negative regulatory mechanisms across recipients and allograft, affecting both the recipient's response and the underlying clinical conditions.^[31,34,35]^ Consistent with these controversial results, our findings still suggested further efforts should be taken to promote the efficacy and utility of QuantiFERON-CMV test in kidney transplantation.

To be noted, it is often to observe the coexistence of CMV infection with the EBV (Epstein–Barr Virus (EBV) infection, and published reports showed the presence of cross-reactivity of EBV-specific IgM antibodies with CMV antigens, indicating that the EBV infection should be a crucial confounding factor of CMV-pp65/IE-1 and QuantiFERON-CMV tests.^[36]^ Unfortunately, none of the included studies further explored the potential influence of EBV coinfection with CMV on the predictive efficacy. Furthermore, posttransplant lymphoproliferative disease (PTLD), mainly caused by EBV infection, required the additional intervention of intravenous immune globulin (IVIG), which inevitably contributed to the disturbances of the allo-immune responses, and then influenced the predictive efficacy of CMV-pp65/IE-1 and QuantiFERON-CMV assays.^[37,38]^ For the improvement of the CMV-immune monitoring assays in kidney transplantation, great considerations should be taken to distinguish CMV infection with potential EBV infection.

Our study has several potential limitations. First, limited case numbers across included studies hindered our further exploration of the CMV-specific immune monitoring, such as subgroup analysis based on the donor and/or recipient CMV status. We hope to update our systematic review and meta-analysis in future, to account for novel studies that will emerge on the CMV ELISPOT and QuantiFERON-CMV tests. Then, we identified several sources of heterogeneity, including testing methods. However, some potential sources, such as selection bias, were ignored due to the lack of extracted information. Finally, the predictive efficacy of these tests during the administration of prophylactic treatments on the risk of CMV DNA viremia after the treatment still needed to be explored.

In conclusion, our systematic review and meta-analysis indicate that CMV-ELISPOT assays, including CMV-pp65 and IE-1, are potentially useful diagnostic tool for the prediction of CMV infection following kidney transplantation. On the contrary, our results suggested that great cautious should be taken for the use of the QuantiFERON-CMV test. Some contributions to promote the efficacy to predict CMV-specific T-cell immunity are urgent. Larger studies are needed to further characterize the utility of CMV immune monitoring system in clinical practice.

## Author contributions

**Conceptualization:** Tianli Niu.

**Data curation:** Yashi Ruan, Wei Guo.

**Formal analysis:** Wei Guo.

**Funding acquisition:** Yashi Ruan.

**Investigation:** Tianli Niu.

**Methodology:** Yashi Ruan, Sudong Liang.

**Project administration:** Yashi Ruan, Sudong Liang.

**Resources:** Sudong Liang, Zhen Xu.

**Software:** Zhen Xu.

**Supervision:** Zhen Xu.

**Visualization:** Zhen Xu, Tianli Niu.

**Writing – original draft:** Yashi Ruan.

**Writing – review and editing:** Yashi Ruan.

## Supplementary Material

Supplemental Digital Content
