# Supplementary material for: Diagnostic performance of cytomegalovirus (CMV) immune monitoring with ELISPOT and QuantiFERON-CMV assay in kidney transplantation: A PRISMA-compliant article
Source: Medicine (Baltimore). 2019 Apr 19;98(16):e15228. doi: 10.1097/MD.0000000000015228 (PMC6494277; doi:10.1097/MD.0000000000015228)

**Supplemental Table 1. Results of the multivariable meta-regression model for the characteristics in included studies.**

| **Variable** | **Coefficient** | **SE** | **P value** | **95%CIs** |
| --- | --- | --- | --- | --- |
| Methods | 0.94 | 0.35 | **0.017** | 1.21, 5.42 |
| ethnicity | 0.19 | 0.57 | 0.74 | 0.36, 4.12 |

Abbreviations: SE, standard error; RDOR, relative diagnostic odds ratio; 95%CIs, 95% confidential intervals.

Supplemental Figure 1: Results of diagnostic meta-analysis for overall CMV immune monitoring in kidney transplantation, including the positive likelihood ratio of overall tests (A), negative likelihood ratio of overall tests (B), positive likelihood ratio of CMV-pp65 (C), negative likelihood ratio of CMV-pp65 (D), positive likelihood ratio of CMV-IE-1 (E), negative likelihood ratio of CMV-IE-1 (F).


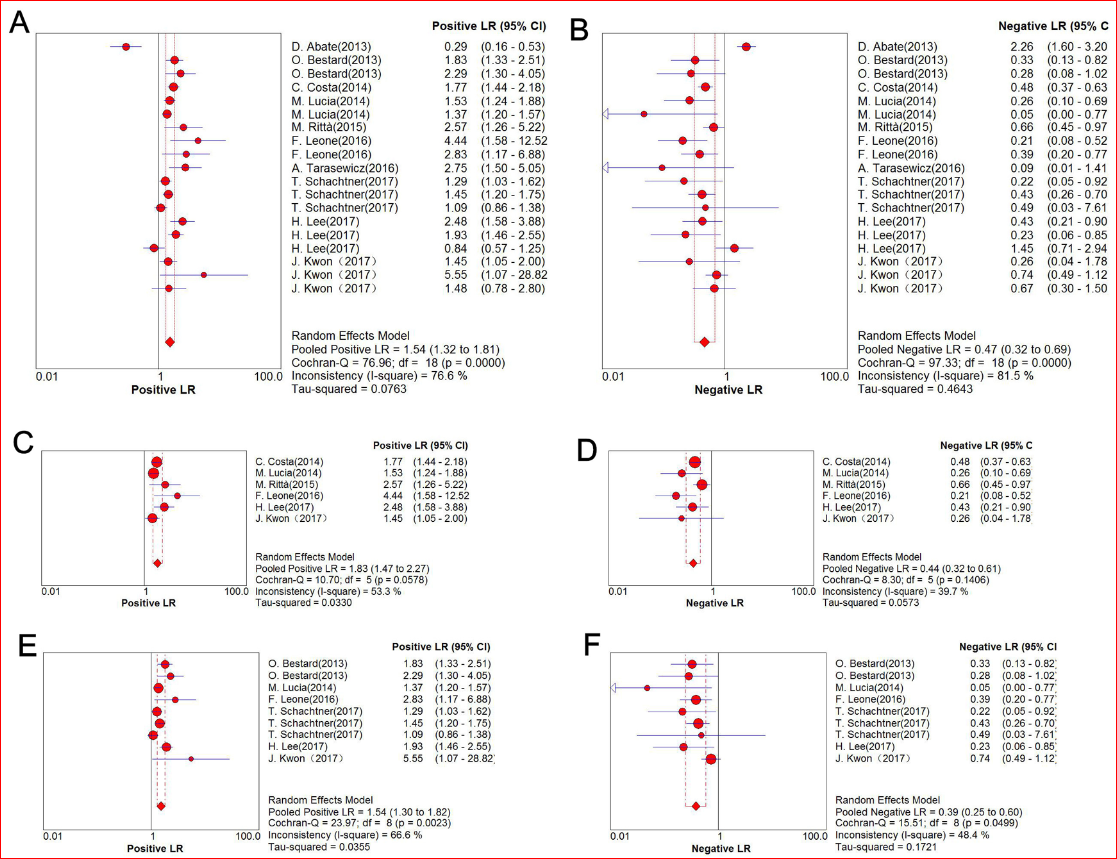


Supplemental Figure 2: Results of predictive meta-analysis for QuantiFERON-CMV test in kidney transplantation, including the pooled sensitivity (A), specificity (B), positive likelihood ratio (C), negative likelihood ratio (D), diagnostic odds ratio (E) and sROC curve (F).


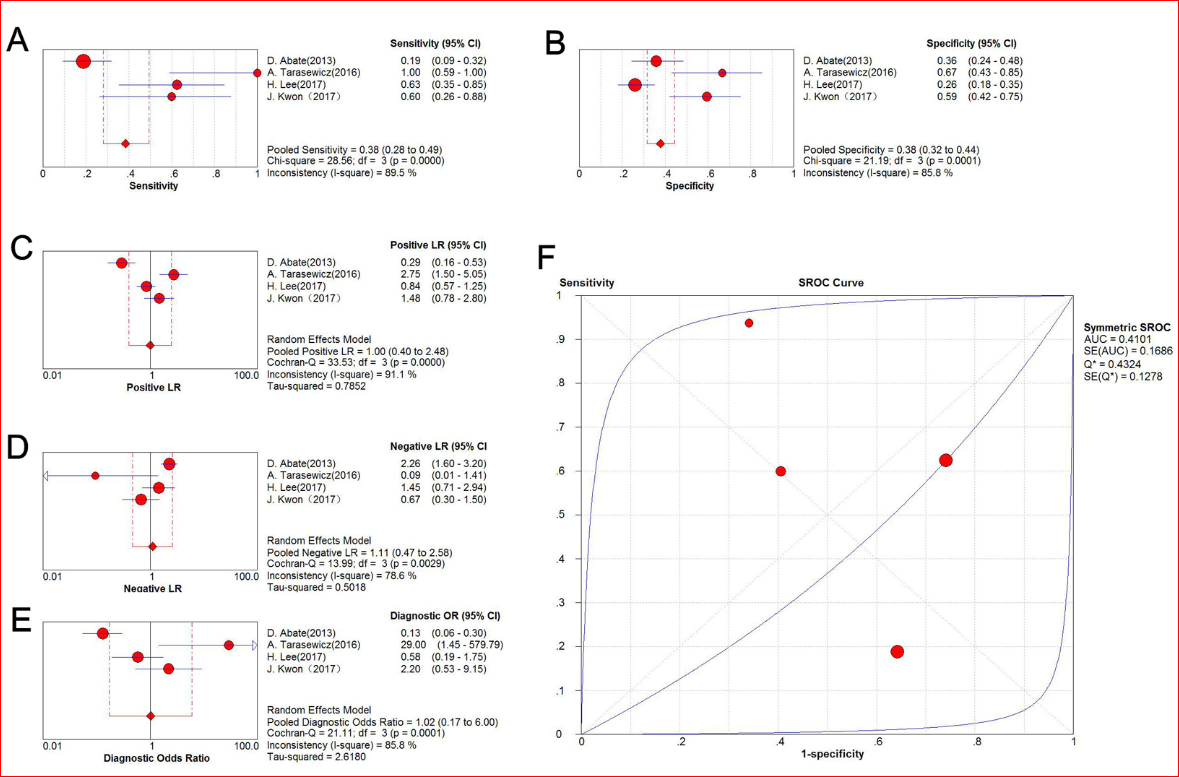

Supplement: Supplemental Digital Content [file medi-98-e15228-s001.doc]
